# Identifying Consumer Groups and Their Characteristics Based on Their Willingness to Engage with Cultured Meat: A Comparison of Four European Countries

**DOI:** 10.3390/foods11020197

**Published:** 2022-01-12

**Authors:** Anouk Boereboom, Philippe Mongondry, Luis K. de Aguiar, Beatriz Urbano, Zheng (Virgil) Jiang, Wim de Koning, Frank Vriesekoop

**Affiliations:** 1 Department of Food, Land and Agribusiness Management, Harper Adams University, Newport TF10 8NB, Shropshire, UK; aj.boereboom@gmail.com (A.B.); ldeaguiar@harper-adams.ac.uk (L.K.d.A.); Virgiljiangzheng@163.com (Z.J.); Wim.deKoning@lincoln.ac.nz (W.d.K.); 2 USC 1422 GRAPPE, INRAE, Ecole Supérieure d’Agricultures, SFR 4207 QUASAV, 49000 Angers, France; p.mongondry@groupe-esa.com; 3 Department of Agricultural and Forestry Engineering, University of Valladolid, 47002 Palencia, Spain; beatriz.urbano@uva.es; 4 Faculty of Agribusiness and Commerce, Lincoln University, Lincoln 7647, New Zealand

**Keywords:** cultured meat, willingness to engage, consumer behaviour, food neophobia, psychographic factors

## Abstract

Cultured meat, as a product of recent advancement in food technology, might become a viable alternative source of protein to traditional meat. As such, cultured meat production is disruptive as it has the potential to change the demand for traditional meats. Moreover, it has been claimed it can be more sustainable regarding the environment and that it is, perhaps, a solution to animal welfare issues. This study aimed at investigating associations between the consumer groups and demographic and psychographic factors as well as identifying distinct consumer groups based on their current willingness to engage with cultured meat. Four European countries were studied: the Netherlands (NL), the United Kingdom (UK), France (FR) and Spain (ES). A sample of 1291 responses from all four countries was collected between February 2017 and March 2019. Cluster analysis was used, resulting in three groups in the NL and UK, and two groups in FR and ES. The results suggest that Dutch consumers are the most willing to engage with cultured meat. Food neophobia and food technology neophobia seem to distinguish the groups the clearest. Moreover, there is some evidence that food cultural differences among the four countries seem to be also influencing consumers’ decision.

## 1. Introduction

Reducing the reliance of growing livestock and as such, reducing meat consumption, is widely acknowledged as a means to mitigate climate change [1,2,3,4]. Cultured meat is an alternative source of animal-based proteins to traditional meat obtained from growing livestock. Cultured meat involves the in-vitro cultivation of skeletal muscle stem cells using a bioreactor [5,6]. It has the potential to become a viable alternative to traditional livestock meat and, as such, alter the production and demand of meat in the future. One argument for supporting cultured meat would be that it could be more sustainable, thus reducing the considerable need for land to produce food to cater to a growing world population. Cultured meat production would also solve issues of animal-transmitted diseases and animal welfare. However, the American Meat Science Association indicated that cultured meat does not yet meet the definition of livestock meat in terms of taste, smell and texture [7]. Furthermore, the actual extent of these benefits is debated [8,9]. Invaluable to the success of any new paradigm-shifting food concept, including cultured meat, is to assess the extent to which consumers would accept it as a source of protein and adopt it in their diet. Consequently, the willingness to accept and engage with cultured meat in different countries has been at the centre of various studies in recent years [10,11,12,13].

Siegrist and Hartmann [13] carried out a large international study and concluded that feelings about uncertainty towards novel foods, product naturalness and disgust were the main factors influencing the acceptance of cultured meat. Bryant and Barnett [14] also highlighted the extent to which cultured meat was perceived as unnatural, which, consequently, raised concerns of unnaturalness acting as a barrier to the acceptance of cultured meat. Studies suggest that men, younger people and meat-eaters are more accepting of cultured meat compared with women, older people and vegetarians/vegans [15,16,17]. Similarly, it has been suggested that consumers in more affluent countries are more accepting of cultured meat than those from less affluent countries [14]. Therefore, for consumer preference and acceptance research involving different countries, the sociodemographic and psychographic factors influencing acceptance for each of the country’s situation are important.

This study is aimed at profiling consumers in four countries based on their willingness to engage with cultured meat, and investigate how countries’ populations differed from each other in their attitudes towards cultured meat. The definition for willingness to engage used in this study will be explained in the Materials and Methods section. Based on the factors influencing the acceptance of cultured meat described in existing research (differences between countries, sociodemographic and psychographic factors), four research objectives were defined:

1. Identify distinct consumer clusters based on their willingness to engage with cultured meat in four European countries: The Netherlands (NL), the United Kingdom (UK), France (FR) and Spain (ES).
2. Investigate associations between the consumer clusters and sociodemographic measures with regard to gender, age, the level of meat avoidance, intended meat consumption and sources of food.
3. Investigate associations between the consumer clusters and psychographic measures such as food (technology) neophobia, the importance of health, the environment and the love for cooking.
4. Determine the outlook for the future of cultured meat for each of the four countries.

The results of this study are aimed at contributing to the understanding of the differences between consumers with regard to their attitudes towards cultured meat.

## 2. Materials and Methods

### 2.1. Data Collection

An extensive questionnaire was initially written in English and subsequently translated by native speakers (to ensure accuracy and preserve its original meaning) into Dutch, French and Spanish. The questionnaire was designed with the objective of gathering extensive information on people’s intentions and perceptions regarding food in general and four alternative protein sources. The survey consisted of distinct groups of statements regarding a specific topic, presented on a five-point Likert Scale, ranging from “Strongly Disagree” to “Strongly Agree” [1→5] or from “Strongly Agree” to “Strongly Disagree” [5→1], depending on the positive or negative nature of the statement [18]. The topics in the questionnaire will be discussed in Section 2.3. of this article. The questionnaire was created using Online Surveys (JISC), formally known as Bristol On-line. A weblink to the questionnaire was sent via e-mail to in-country contacts who were responsible for distributing it among pre-existing contact lists, which yielded most of the responses. Social media platforms such as LinkedIn were also used [18,19]. All data was collected, collated and centrally stored at the Harper Adams University server in the UK. The questionnaire was approved by Harper Adams University Research Ethics Committee.

A total sample of 1291 responses across the four countries was collected between February 2017 and March 2019. The answers were coded and analysed in IBM SPSS version 26. The sociodemographic profile of the sample from the four countries can be seen from Table 1.

### 2.2. Cluster Analysis

To identify separate segments of respondents based on their willingness to engage with cultured meat, a series of two-step cluster analyses in IBM SPSS Statistics version 26 were carried out for each country, without specifying a maximum or fixed number of clusters, thus, to determine as many consumer clusters from the data as possible. Cluster membership variables were created as output i.e., a respondent’s cluster membership was determined by their willingness to engage with cultured meat. The respondents’ willingness to engage was defined by their answers to four questions that were included in the questionnaire: “If available, would you consider cultured muscle tissue as a source of dietary protein?”, “Would you personally be willing to try cultured meat?”, “Would you personally be willing to purchase cultured meat?” and “Would you personally be willing to pay more for cultured meat?”

These four variables were used as input for cluster analysis to create the clusters. Similar variables have been used in existing literature investigating willingness to engage with cultured meat: Bryant et al. [10,20] studied willingness to try and purchase cultured meat measuring acceptance. Yet, Zhang et al. [6] concluded that acceptance influenced purchasing behaviour. Thus, they conceptualised purchasing behaviour by using the willingness to try and purchase as separate from acceptance. Furthermore, when measuring engagement with cultured meat, Liu et al. [12] examined the willingnesses to try, purchase and pay for cultured meat individually. This variability in measurements used to determine acceptance and willingness to engage can lead to different results and makes comparative analysis difficult [21].

### 2.3. Tested Variables

Sociodemographic measures were used to test associations such as gender, age, level of current meat avoidance (Table 1), and intended meat consumption in the future (possible answers: “I intend to decrease the amount of meat I consume”, “I intend to consume the same amount of meat this time next year as I do now” or “I intend to increase the amount of meat I consume”). Two statements about the sources of food (“I often grow my own vegetables and/or herbs” and “I often get my food from traditional and/or local sources”) were added to the analysis to further specify consumers’ characteristics in each cluster.

The questionnaire also contained the statements of The Food Neophobia Scale (FNS). The FNS is a tested and validated scale to measure reluctance to engage with novel foods [19,22]. It consists of five neophiliac statements and five neophobic questions. In addition, statements from the Food Technology Neophobia scale (FTNS), proposed by Cox and Evans [23] were also included. The FTNS measures fear of novel food technologies. The higher the score on these two scales, the more food (tech) neophobic a respondent is.

Psychographic measures that were tested included: ‘*the importance of healthiness of food choices*’ [18,24] ‘*the love for cooking*’ [18,25], and ‘*attention for the environmental when making food choices*’ [18,26,27]. Table 2 shows the groups of statements belonging to each measure. The higher the mean score for each measure, the more important that factor is for a person when making food choices.

To determine attitudes towards the future of cultured meat, answers to the question “Do you think that dietary cultured muscle tissue derived proteins provide a realistic alternative to offset a growing demand for animal-based proteins?” were analysed for each country. Possible answers were: “It is a fad”, “it is available now”, “it will be available in the short term (2030)” or “it will be available in the long term (2050)”. No statistical tests were carried out for this measure.

### 2.4. Statistical Analysis

To create consumer clusters (objective 1), a two-step cluster analysis was used, with Schwarz’s Bayesian Criterion as a clustering criterion and log-likelihood as a distance measure to determine the number of clusters. Chi-square tests were performed between the clusters created and the four categorical variables (described in Section 2.2) to determine if the clusters were significantly different.

Associations between the clusters and sociodemographic measures (objective 2) were tested by applying Chi-Square tests for goodness-of-fit with adjusted residuals for pairwise comparison, which were performed to compare proportions of the number of people in each cluster.

Before testing associations between the clusters and psychographic measures (third objective), preliminary confirmatory principal component analysis (PCA) using Varimax with Kaiser normalization as the rotation method was carried out for each measure to confirm that the groups of statements for each measure were strongly enough related to be collapsed into one score and simplify the analysis by reducing dimensions for each measure to avoid multicollinearity.

Statistical tests to investigate associations between the clusters and psychographic measures were chosen based on the distribution of the measure and the number of clusters in each country to evaluate the distribution of a measure, Shapiro–Wilk tests for small samples were performed. Based on the non-Gaussian distribution of most of the test variables and the qualitative nature of the data, Kruskal–Wallis tests were used to test for differences. Significant results were adjusted by the Bonferroni correction for pairwise comparison, to determine the source of the significance.

## 3. Results

### 3.1. Identifying Consumer Clusters (Objective 1)

**Figure 1:**
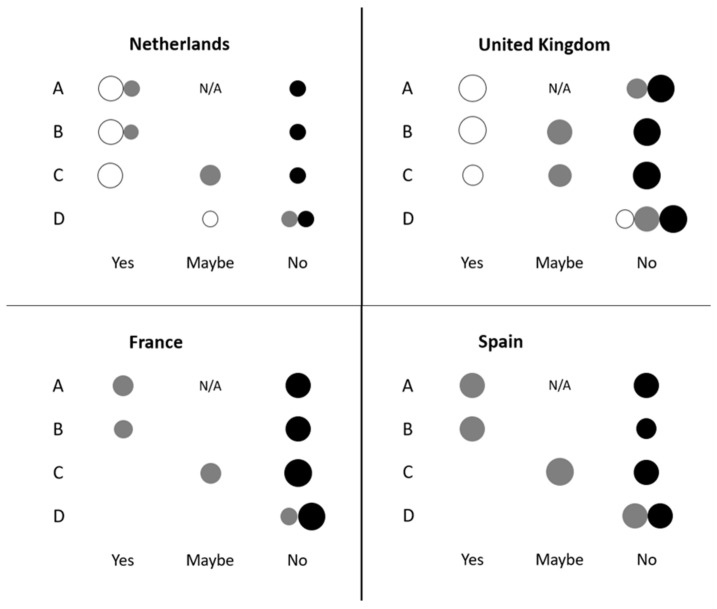
Cluster analyses with regards to willingness to engage with cultured meat showing the spread among each of the four categorigal variables. The size of the bubbles is indicative of the relative distribution among respondents within each variable. The categorical variables are: A, “If available, would you consider cultured muscle tissue as a source of dietary proteins?”; B, “Would you personally be willing to try cultured meat?”; C, “Would you personally be willing to purchase cultured meat?” and D, “Would you personally be willing to pay more for cultured meat?”. The colours of the bubbles represent the clusters: white = yes, willing, grey = uncertain, black = no, not willing. France, *n* = 484; Spain, *n* = 210; The Netherlands, *n* = 230; and The United Kingdom, n = 366. N/A and all blanks= no association.

In both NL and the UK, three distinct clusters were identified: ‘Willing’, ‘Uncertain’ and ‘Not Willing’ (Table 3). The names of the clusters reflect the willingness to engage with cultured meat of the respondents in each of the clusters. In both FR and ES two clusters were identified: ‘Uncertain’ and ‘Not Willing’. Based on the answers to the four variables used to create the clusters, no ‘Willing’ cluster could be identified in either FR or ES. Chi-square tests were performed (*α* = 0.05, *p* < 0.001 in all countries) which confirmed that the clusters were significantly different. Figure 1 presents a bubble graph for each country. Each bubble graph shows how respondents from each cluster (the clusters are indicated in Figure 1 by the colours white, grey and black) answered the four questions that were used as input for the cluster analysis (described in Section 2.2, and indicated in Figure 1 by the letters A, B, C and D). The size of the bubbles is indicative of the distribution of respondents within each variable. Based on this spread, the attitude towards cultured meat (presented in Table 3) was determined.

### 3.2. Confirmatory PCA Results

Preliminary PCA was carries out to justify collapsing the statements from each measure into one score. The Kaiser–Meyer–Olkin Measure of Sampling Adequacy (created as output of the PCA), indicating the proportion of variance explained by the underlying statements, should be >0.6, and Bartlett’s test for sphericity should be significant at *p* < 0.05 [28]. This was confirmed for the measures ‘*importance of healthiness of food choices*’ (*df = 3)* and ‘*the love for cooking*’ (*df* = 6) explaining >63% of the variance in each country. For the measure ‘*attention for the environmental when making food choices*’ (*df* = 10), the KMO scores were <0.6 for the FR and ES (0.578 and 0.539, respectively). Total % of variance explained was 44% and 45% in NL and UK, respectively, and 57% and 62% in FR and ES. As the score (0.672) was not drastically below 0.7, it was chosen to keep the measure in the study. Total variance explained was also relatively low in NL and UK, so caution was taken in assessing the results associated with this variable. *p*-values for all three variables in all four countries were *p* < 0.001.

### 3.3. Associations between the Clusters and Sociodemographic Measures (Objective 2)

No associations were found between the clusters and respondents’ age or gender in NL nor FR. In the UK, men were more likely to be in the W cluster (54%) than women (29%) (*χ*^2^ = 20.90, *df* = 2, *p* < 0.001). In ES, Kruskal–Wallis tests revealed respondents in the U cluster were more likely to be older (*µ* = 38) than in the NW cluster (*µ* = 33) (*χ*^2^(1) = 5.262, *p* = 0.022).

Chi square tests revealed associations between the clusters and level of meat and animal product avoidance in the NL data (*χ*^2^ = 19.39, *df* = 4, *p* = 0.001) and UK data (*χ***^2^** = 44.04, df = 4, *p* < 0.001). NL and UK respondents who avoid meat or animal products completely were more likely to be in the NW cluster (40% and 64% respectively). NL respondents who occasionally avoid meat or animal products were more likely to be in the W cluster (56%). In the UK, respondents who eat meat were more likely to be in the W cluster (41%).

Chi-square tests revealed an association between a respondent’s future meat consumption and the clusters in the NL (*χ*^2^ = 27.06, *df* = 2, *p* < 0.001). NL respondents who intended to decrease their meat consumption were more likely to be in the W cluster (62%) For FR and ES, no associations were found between the clusters and a respondent’s future meat consumption.

The only country with significant results on the two questions about local sources and growing vegetables was FR. Kruskal–Wallis test results revealed respondents in the NW cluster were more likely to grow their own vegetables (*χ*^2^(1) = 8.700, *p* = 0.003, *µ* = 2.89) and more likely to get their food from local sources (*χ*^2^(1) = 20.049, *p* < 0.001, *µ* = 3.84) than respondents in the U cluster (*µ* = 2.52 and *µ* = 3.42 respectively).

### 3.4. Associations between the Clusters and Psychographic Measures (Objective 3)

No significant associations were found between the clusters and the measures: ‘*importance of health*’, ‘*love for cooking*’. However, for the measure *‘importance of the environmental*’, an association was found in NL (*χ*^2^(2) = 6.463, *p* = 0.040): respondents in the W cluster (*µ* = 3.85) deemed the environmental more important than respondents in the U cluster (*µ* = 3.58).

Associations were found between food neophobia and the clusters in NL, UK, and FR (Table 4). Dutch respondents in the W cluster and U cluster scored significantly lower than those in the NW cluster. UK respondents in the W cluster scored lower than those in the NW cluster. FR respondents in the U cluster scored significantly lower than those in the NW cluster. No associations were found between food neophobia and the clusters in ES.

Associations between the food technology neophobia and the clusters were found in all countries (Table 4). UK respondents in the W cluster scored significantly lower than respondents in the U cluster and NW cluster. FR respondents in the NW cluster scored significantly higher than the U cluster. No associations were found between food technology neophobia and the clusters in ES.

### 3.5. The Future of Cultured Meat (Objective 4)

**Figure 2:**
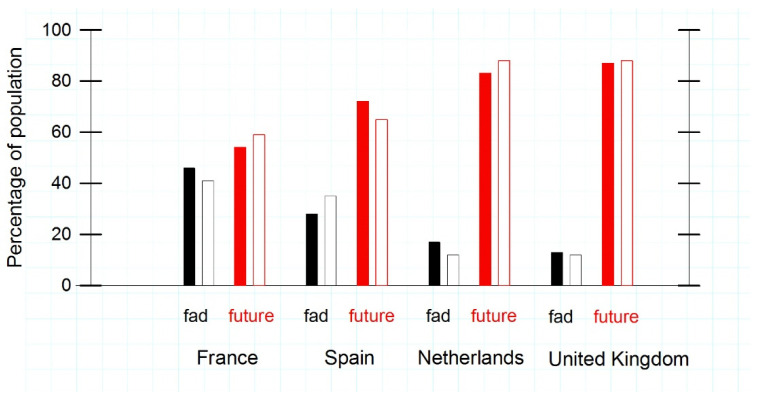
Does cultured meat have a future? Percentage of people’s (by gender) in each country in response to the question: “Do you think that dietary cultured muscle tissue derived protein provide realistic alternative to offset a growing demand for animal-based proteins?”. France, *n* = 484; Spain, *n* = 210; The Netherlands, *n* = 230; and the United Kingdom, *n* = 366. The solid black bars represent males who believe that cultured meat is a fad, white bars (black outline) represent females who believe that cultured meat is a fad, red bars represent males who believe that cultured meat has a future, and the white bars with red outlines represent females who believe that cultured meat has a future. Per gender, the two bars for each answer possibility (fad or future) together form 100% in each country. Chi-square tests were performed for each country, which revealed no significant association between gender and the opinion about the future of cultured meat. The data presented was normalised in order to compare the data to the same scale.

We assessed the notion of whether cultured meat present a realistic alternative to livestock proteins by posing the following question: “Do you think that dietary cultured muscle tissue derived proteins provide a realistic alternative to offset a growing demand for animal-based proteins?”, respondents could choose between four possible answers. Out of the four possible answers, the three answers indicating that cultured meat has a realistic future were merged into one in order to create greater clarity regarding the view of the respondents’ perception of cultured meat into the future (Figure 2). The results are presented by gender, as to account for the unequal proportion of males and females in each country. Respondents who preferred not to disclosure their gender were taken out of this analysis. Chi-Square tests performed in each country revealed no significant association between gender and one’s opinion about the future of cultured meat. The largest percentages of males and females who thought cultured meat represents a fad were found in France, followed by Spain; while participants from the UK and The Netherlands had the most positive outlook with regard to a realistic future for cultured meat (Figure 2).

## 4. Discussion

Utilising cluster analysis has been proven to be an appropriate method of identifying consumer groups in the data, which is supported by the literature on consumer groups regarding food choices and preferences [29,30,31]. The initial clustering of consumers revealed differences between the four countries analysed in this study. The NL and UK both had a cluster with consumers showing a relatively high willingness to engage with cultured meat, although such a cluster was larger in the NL than in the UK (Figure 1). FR and ES did not reveal a cluster deemed to be very willing to engage with cultured meat. In ES the largest cluster was the U cluster, which suggests consumers could still be persuaded to engage with cultured meat in the future. This agrees with the findings of Francekovic et al. [11] who stated that consumers in Spain were likely to buy cultured meat if that was affordable, and by Gómez-Luciano et al. [18] who found Spanish consumers to be even more accepting (42%) than UK consumers (20%). In this study, based on the cluster analysis, Dutch consumers were the most willing to engage with cultured meat, followed by those in the UK. These findings agree with what Grasso et al. [16] reported in a study among older consumers. One of the factors explaining the high willingness in the NL could be attributed to positive media coverage on cultured meat outweighing negative media coverage [31,32] as well as the ongoing research being conducted there [5,21,33,34,35]. It has also been shown that the level of exposure to an alternative protein source influences the willingness to engage with it [36]. This perhaps partly explains the reason Spanish respondents showed a lower willingness to engage with cultured meat compared to NL and UK, since the awareness about cultured meat is relatively low in that country [11].

Awareness about cultured meat, not only as a product, but also about the current meat production system, seems to play an important role in the willingness to engage with cultured meat. In previous studies, consumers working in meat or livestock production were found to be more willing to try and purchase cultured meat. That may be due to their knowledge about the industry, therefore their wish to looking for alternatives [12,21]. Awareness in general can lead to higher willingness to reduce meat consumption, which, consequently, positively influences willingness to engage [17,37]. This study found that those consumers looking to reduce their meat consumption were also more willing to engage with cultured meat.

In France, the largest cluster was the NW cluster, thus indicating the French consumers to be the least willing to engage with cultured meat. While exploring food culture was not part of the objectives of this study, cultural traits regarding food could be influencing consumers’ willingness to engage with cultured meat as was also suggested by Hartmann and Siegrist [13] among their French cohort.

The Dutch consumers had the most positive attitude with regards to the future of cultured meat; while almost half of the French respondents considered cultured meat to be a fad. Food traditions and heritage are imperative to French consumers [13]. Therefore, it could be said that since the French opinion about cultured meat is entrenched, as evidenced by expressing negatively about cultured meat on multiple aspects in our study and finding lower acceptance rates compared to other countries in previous studies [10,13], only more exposure could perhaps improve the French perception towards cultured meat over time [36]. Nevertheless, the results from this study suggest that FR is not the country where commercial cultured meat would be rolled out in Europe. Despite this, Bryant et al. [10] concluded France could still be a viable market for cultured meat, mainly because of the large portion of meat-eaters looking to reduce their meat consumption, a result that could not be confirmed by our current study.

The results from the cluster analysis in this study suggest a split in opinions between NL and UK on the one (more willing) side, and FR and ES on another (less willing) side. Despite the four aforementioned countries having not been previously studied regarding the willingness to accept cultured meat, there were similarities in the respondents’ outlooks (e.g., seen in Figure 2). For example, in Belgium, Bryant and Sanctorum [15] found the northerners, in the Flemish-speaking part of Belgium, to be more willing than the French-speaking southerners. Furthermore, Germans tended to be more accepting than the French [10]. Differences in diet and values surrounding food might underpin these cultural differences, an aspect of consumption which has been underexplored thus far. In addition, Bryant and Barnett [14] and Gómez-Luciano et al. [18] also found that consumers in lower income countries were less open to cultured meat. In 2019, the NL had the highest gross domestic product (GDP) per capita (US$52,331), followed by the UK (US$42,330), FR (US$40,494) and ES (US$29,600) [38]. These figures roughly correspond to the willingness to engage with cultured meat in The NL which has the highest willingness (based on the cluster analysis), followed by the UK. However, despite the ES having a lower DGP per capita than FR, the respondents demonstrated to have a higher willingness to engage cultured meat than FR.

Although the use of cluster analysis enabled identifying different consumers’ groups, clear consumers’ profiles showing well-defined characteristics was not possible. On the one hand, sociodemographic characteristics were only significant for the ES case. On the other hand, only age and gender were significant in France. This has proven insufficient to create a more well-defined profile. An explanation for this could be attributed to cultured meat not yet being available to consumers, which could become clearer when such a product reaches the market.

As for the psychographic factors of food neophobia and food technology neophobia, these seem to distinguish the groups the most. This study found that higher levels of food (tech) neophobia resulted in a lower willingness to engage with cultured meat. This is in line with previous studies on food neophobia and alternative protein sources [10,11,18,37]. The other factors investigated, such as the ‘importance of the environment’ and ‘health and cooking’ were not distinguishing enough. This could mean these factors were still not directly linked with willingness to engage with cultured meat, as the respondents have not yet fully experienced it, as suggested by Gómez-Luciano et al. [18].

## 5. Limitations

As mentioned before (footnote Table 1), for the individual countries, the sample populations used in this study provide a confidence level of 95%, with margins of error varying from 4.5% to 6.7%. However, bearing in mind the size of the combined-country clusters the margin of error is 4% or lower at a 98% confidence level, which means that the predictions for the clusters and the individual countries can still be made. It is possible that, with a higher number of respondents, more relationships (e.g., between meat consumption and willingness to engage with cultured meat) could have been found. Furthermore, there is a risk of sample selection-bias, because the survey was distributed though social networks and pre-existing lists. The convenience sampling technique that was used to collect the data, relies on the willingness of people to spend a relatively long time (15 min) to fill in the survey that was used for this study. However, Gómez-Luciano et al. [18] argued that using a different sampling technique, such as paying respondents to participate, does not necessarily result in higher-quality responses, because the participants do not have affection with the topics. The unequal distribution of the genders in the samples creates difficulty for the generalisation of the findings. For example, women were overrepresented in the FR sample (81%). Considering that women seem less accepting of cultured meat [15,18] this could explain the higher number of people found that considered cultured meat to be a fad (Figure 2). Lastly, because cultured meat is not widely available to current consumers, our findings are based on the intentions of people towards a product that does not exist yet. The actual taste and price of cultured meat when it becomes available will likely influence the acceptance of the product [11,17] Therefore, it is recommended to repeat this study when cultured meat becomes more widely available to the public, in order to confirm or refute the results from the current study.

## 6. Conclusions

Overall, this study found that the consumers who would be the most willing to engage with cultured meat to be those who are currently meat eaters but who would like to decrease their meat consumption. These also tended to have a relatively low fear for new or unknown food products as well low or no fear of new food technologies. Of the four countries studied, the largest group of consumers willing to accept cultured meat was found in The Netherlands, followed by the United Kingdom. Although meat eaters who are willing to decrease their meat consumption in Spain currently represent a small group, there seems to be an opportunity for this group to become larger should positive exposure and awareness to cultured meat increase. French consumers were the least willing to engage with cultured meat. Future research into the willingness to engage with cultured meat should explore the influence of cultural determinants, as they seem to underpin differences found between the studied countries.

## Figures and Tables

**Table 1:** Sociodemographics of the respondents in the four countries.

		The Netherlands	UK	France	Spain
		*n* = 231 *	*n* = 366	*n* = 484	*n* = 210
gender	male	37.8%	23.8%	18.6%	50.7%
	female	62.2%	76.2%	81.4%	49.3%
age	<24	54.5%	44.0%	57.6%	34.3%
	25–49	30.3%	42.6%	30.2%	48.6%
	50–64	14.7%	12.0%	11.6%	10.0%
	65+	0.4%	1.4%	0.6%	7.1%
Meat avoidance	Yes. I do not consume meat or animal products	16.5%	17.5%	8.3%	3.3%
	Not really, but I specifically avoid meat or animal products on some days	41.1%	15.8%	31.8%	34.8%
	No	42.4%	66.7%	59.9%	61.9%

* The country-by-country sample sizes for the purpose of a study of this nature could be considered small. However, considering the populations in those the individual countries, at a confidence level of 95%, the margin of error varies from 4.5% to 6.7%. While at a confidence level of 90%, the margin of error varies from 3.7% to 5.6%. Therefore, the conclusions drawn from this study still represent a reasonable alignment with the individual countries. Margins of error were calculated using the standard deviation of the population (σ), the sample size (*n*) and the *z*-score of the confidence interval (for 95% = 1.960, for 98% = 2.326): z * (σ/√*n*).

**Table 2:** Statements belonging to each psychographic measure.

Measure	Statements
importance of health	1. The healthiness of food has little impact on my food choices.
	2. I am very particular about the healthiness of the food I eat.
	3. I eat what I like, and I do not worry much about the healthiness of the food.
love for cooking	1. The less I have to do to prepare a meal-the better.
	2. I love cooking and will spend a lot of time and effort to prepare foods on a daily basis.
	3. At home, I preferably eat meals that can be prepared quickly.
	4. Even though I live a busy life, whenever possible I love to cook and bake.
importance of the environmental	1. When I buy food, I try to consider how my use of them will affect the environment.
	2. I am worried about humankind’s ability to provide the nutritional needs for all people living on earth now.
	3. Something drastic must change to feed all the people on earth by 2050.
	4. The world can easily sustain the food demands of a growing population in one or two generations.
	5. Humankind is NOT responsible for global warming.

**Table 3:** Summary of the clusters for each country.

		1	2	3
	Cluster Name	Willing (W)	Uncertain (U)	Not Willing (NW)
The Netherlands	*n*	105	85	41
*n* = 231	%	45.5	36.8	17.7
size ratio: 2.56	attitude towards cultured meat	*positive*	*uncertain with a positive trend*	*negative*
United Kingdom	*n*	127	127	112
*n* = 366	%	34.7	34.7	30.6
size ratio: 1.13	attitude towards cultured meat	*positive*	*uncertain with a negative trend*	*negative*
France	*n*	-	201	283
*n* = 484	%	-	41.5	58.5
size ratio: 1.14	attitude towards cultured meat	-	*uncertain*	*negative*
Spain	*n*	-	133	77
*n* = 210	%	-	63.3	36.7
size ratio: 1.73	attitude towards cultured meat	-	*uncertain*	*negative*

**Table 4:** Comparative Kruskal–Wallis * results for FNS and FTNS (*α =* 0.05). *p*-values are significant for *p* < 0.05.

Food Neophobia						Food Technology Neophobia					
Country	*χ* ^2^	*p*-Value	Cluster	Mean	SD **	Country	*χ* ^2^	*p*-Value	Cluster	Mean	SD
NL	14.738	<0.001	W	2.1	0.58	NL	21.961	<0.001	W	2.4	0.50
			U	2.2	0.64				U	2.7	0.67
			NW	2.6	0.63				NW	3.1	0.84
UK	17.730	<0.001	W	2.2	0.71	UK	24.712	<0.001	W	2.6	0.47
			U	2.3	0.57				U	2.9	0.48
			NW	2.6	0.63				NW	3.0	0.63
FR	7.539	0.006	U	2.2	0.64	FR	22.548	<0.001	U	3.1	0.51
			NW	2.4	0.48				NW	3.3	0.55
ES	0.170	0.694	U	2.3	0.66	ES	3.755	0.053	U	3.1	0.69
			NW	2.3	0.58				NW	2.9	0.56

* degrees of freedom: NL and UK = 2, FR and ES = 1. ** SD: standard deviation; France, *n* = 484; Spain, *n* = 210; The Netherlands, *n* = 230; and the United Kingdom, *n* = 366.

## Data Availability

The data presented in this study are available on request from the corresponding author.
